# The IL-13/IL-4R*α* axis is involved in tuberculosis-associated pathology

**DOI:** 10.1002/path.4399

**Published:** 2014-08-06

**Authors:** Lisa Heitmann, Mahin Abad Dar, Tanja Schreiber, Hanna Erdmann, Jochen Behrends, Andrew NJ Mckenzie, Frank Brombacher, Stefan Ehlers, Christoph Hölscher

**Affiliations:** 1 Infection Immunology, Research Centre BorstelGermany; 2 Priority Research Area ‘Infections’, Research Centre BorstelGermany; 3 Fluorescence Cytometry, Research Centre BorstelGermany; 4 MRC Laboratory of Molecular BiologyCambridge, UK; 5 International Centre for Genetic Engineering and Biotechnology, University of Cape TownObservatory, South Africa; 6 Molecular Inflammation Medicine, Christian-Albrechts-UniversityKiel, Germany; 7 Cluster of Excellence ‘Inflammation at Interfaces’ (Borstel–Kiel–Lübeck–Plön), Christian-Albrechts-UniversityKiel, Germany; 8 German Centre for Infection ResearchBorstel, Germany

**Keywords:** tuberculosis, pathogenesis, mice, cytokines, macrophages

## Abstract

Human tuberculosis (TB) is a leading global health threat and still constitutes a major medical challenge. However, mechanisms governing tissue pathology during post-primary TB remain elusive, partly because genetically or immunologically tractable animal models are lacking. In human TB, the demonstration of a large relative increase in interleukin (IL)-4 and IL-13 expression, which correlates with lung damage, indicates that a subversive T helper (TH)2 component in the response to *Mycobacterium tuberculosis* (*Mtb*) may undermine protective immunity and contribute to reactivation and tissue pathology. Up to now, there has been no clear evidence regarding whether IL-4/IL-13-IL-4 receptor-*α* (R*α*)-mediated mechanisms may in fact cause reactivation and pathology. Unfortunately, the virtual absence of centrally necrotizing granulomas in experimental murine TB is associated with a poor induction of a TH2 immune response. We therefore hypothesize that, in mice, an increased production of IL-13 may lead to a pathology similar to human post-primary TB. In our study, aerosol *Mtb* infection of IL-13-over-expressing mice in fact resulted in pulmonary centrally necrotizing granulomas with multinucleated giant cells, a hypoxic rim and a perinecrotic collagen capsule, with an adjacent zone of lipid-rich, acid-fast bacilli-containing foamy macrophages, thus strongly resembling the pathology in human post-primary TB. Granuloma necrosis (GN) in *Mtb*-infected IL-13-over-expressing mice was associated with the induction of arginase-1-expressing macrophages. Indirect blockade of the endogenous arginase inhibitor l-hydroxyarginine in *Mtb*-infected wild-type mice resulted in a strong arginase expression and precipitated a similar pathology of GN. Together, we here introduce an experimental TB model that displays many features of centrally necrotizing granulomas in human post-primary TB and demonstrate that IL-13/IL-4R*α*-dependent mechanisms leading to arginase-1 expression are involved in TB-associated tissue pathology. © 2014 The Authors. *The Journal of Pathology* published by John Wiley & Sons Ltd on behalf of Pathological Society of Great Britain and Ireland.

## Introduction

Granuloma formation is a hallmark of *Mycobacterium tuberculosis* (*Mtb*) infection and represents the histological correlate of inflammatory tissue responses generally associated with protective immunity [1,2]. Disease develops when an initial granulomatous focus cannot fully contain mycobacterial replication. In adult humans this most often occurs when, despite existing adaptive immunity, a previously stably persistent *Mtb* resumes growth within the granuloma. This so-called ‘reactivation’ tuberculosis (TB) is characterized by granuloma necrosis (GN) and subsequent cavity formation, during which the ‘caseous’ centre of necrotized granulomas liquefies and erodes into a bronchus, spreading *Mtb* into the environment. Therefore, reactivation, GN and cavity formation not only contribute to pathology but are also significantly involved in spreading the disease. However, mechanisms governing tissue pathology during post-primary TB remain elusive, partly because genetically or immunologically tractable animal models are lacking.

In contrast to protective T helper (TH)1 cell-mediated immunity 3, the role of an interleukin (IL-4)-/IL-13-driven TH2 immune response for susceptibility to TB is discussed controversially. Experimental models of latent and progressive TB revealed that disease progression after high-dose *Mtb* infection or corticosterone treatment, respectively, was associated with *il4* gene expression 4,5. IL-4-deficient (^–/–^), IL-4 receptor-*α* (R*α*^–/–^) and signal transducer and activator of transcription (STAT)6^–/–^ mice on a C57BL/6 genetic background have been shown to display bacterial loads similar to those of wild-type mice when infected with *Mtb* 6,7. However, in BALB/c mice, high-dose *Mtb* inoculation induced reactivating progressive pulmonary TB that was associated with an elevated TH2 immune response during the late stages of infection 8,9. Accordingly, neutralization of IL-4 in intravenously infected BALB/c animals and high-dose intratracheal *Mtb* infection of BALB/c IL-4^–/–^ mice 6 resulted in decreased bacterial loads and attenuated pathology 10,11. Because TB patients in developing countries are highly exposed to *Mtb* and express an increased TH2 immune response, experimental high-dose infection of BALB/c mice was introduced as a model for TB in poor countries 12,13. Together, data derived from this model so far demonstrate that a TH2 immune response contributes to disease progression, and therefore blocking IL-4 has been proposed as a therapeutic approach 8.

In human TB, the demonstration of a large relative increase in IL-4 and IL-13 expression, which correlates with lung damage, indicates that a subversive TH2 component in the response to *Mtb* may undermine protective immunity and contribute to reactivation and tissue pathology 14,15. On the other hand, a strong TH2 response is not consistently associated with TB disease 16,17. Until now there has been no clear evidence regarding whether high expression of IL-4 and IL-13 is a consequence of disease recrudescence, or whether IL-4R*α*-mediated mechanisms may in fact cause reactivation and pathology. However, the identification of a molecular pathway that directs reactivation, GN and cavity formation would greatly facilitate the development of new therapeutic strategies aimed at preventing or treating post-primary TB.

## Materials and methods

### Mice

IL-4R*α*-deficient (^–/–^) 18 and IL-13-over-expressing (^tg^) mice 19 were on a C57BL/6 and IL-13^tg^ × IL-4R*α*^–/–^ mice on a BALB/c genetic background. Mice were bred under specific-pathogen-free conditions at the Research Centre Borstel or the Max-Planck-Institute for Evolutionary Anthropology, Leipzig, Germany. All experiments performed were in accordance with the German Animal Protection Law and were approved by the Animal Research Ethics Board of the Ministry of Environment, Kiel, Germany.

### Bacteria and aerosol infection

Mice were infected with a low dose of 100 CFU *Mtb* H37Rv, as described previously 20.

### Neutralization of NOS2

To inhibit NOS2, mice received l-*N*6-(1-iminoethyl)-lysine (L-NIL; Alexis, Lörrach, Germany) diluted to 10 mm in drinking water.

### Colony enumeration assay and histology

Bacterial loads in lungs were calculated as described previously 20. Histopathological evaluation, detection of acid-fast bacilli, collagen deposition, immunohistochemical analysis of hypoxia, nitric oxide synthase (NOS)2 and arginase (Arg)-1 were performed as previously described 20,21. A modified protocol was used for immunofluorescent co-staining of *α*-smooth muscle actin (*α*-sma) and Arg-1. After antigen-retrieval in 1% SDS, blocking and incubation with primary antibodies, sections were stained with goat anti-mouse-Alexa488 (Invitrogen, Darmstadt, Germany) and goat anti-mouse-Cy5 (Dianova, Hamburg, Germany), respectively, and DAPI (Roche, Mannheim, Germany). For co-staining of CD3, CD68, tumour necrosis factor (TNF; all from Abcam, Cambridge, UK) and Arg-1 (Santa Cruz, Heidelberg, Germany) cryosections were fixed, blocked and the primary antibody applied. After overnight incubation, the sections were stained with goat-anti-rat-Alexa488 and goat-anti-rabbit-Alexa546 or goat-anti-rabbit-Alexa488 and goat-anti-rabbit-Alexa633, respectively, and DAPI. Fluorescent co-staining was analysed using a TCS SP5 fluorescent microscope and LAS AF software (both from Leica, Wetzlar, Germany). Lipid droplets in cryosections were stained in oil red O solution (Sigma, Munich, Germany) for 20 min after fixation in 10% formalin and 60% 2-propanol. After washing in 60% 2-propanol and water, the sections were counterstained with haematoxylin (Vector, Lörrach, Germany).

### Delayed type hypersensitivity (DTH)

DTH was determined 28 days after infection, as described previously 22.

### Quantitative real-time RT–PCR

Quantitative real-time RT–PCR was performed as described previously 20,23,24.

### Cytokine determination

The concentrations of cytokines in lung homogenates from uninfected and infected mice were determined by CBA (BD Bioscience), as described previously 20.

### Flow cytometry

Restimulation of lung cells from *Mtb*-infected mice, subsequent intracellular cytokine staining in CD4^+^ T cells and flow-cytometric analysis have been described previously 23.

### IFN*γ* ELISPOT assay

Detection of antigen-specific interferon-*γ* (IFN*γ*)-producing CD4^+^ T cells from infected lungs was conducted as described previously 20.

### Determination of arginase activity and collagen deposition

Arginase activity in murine tissue was quantified as previously described 24. The collagen content in lung homogenates was determined using a quantitative dye-binding method designed for the analysis of acid-soluble collagens, as described by the manufacturer (Sircoll™, Biocolor).

### Statistical analysis

Quantifiable data are expressed as mean and standard deviation (SD). After analysing for Gaussian distribution, unpaired Student's *t*-test or the Mann–Whitney test was applied, defining different error probabilities. Statistical survival analysis was performed using the log-rank test.

## Results

### IL-13^tg^ mice develop recrudescent tuberculosis

**Figure 1:**
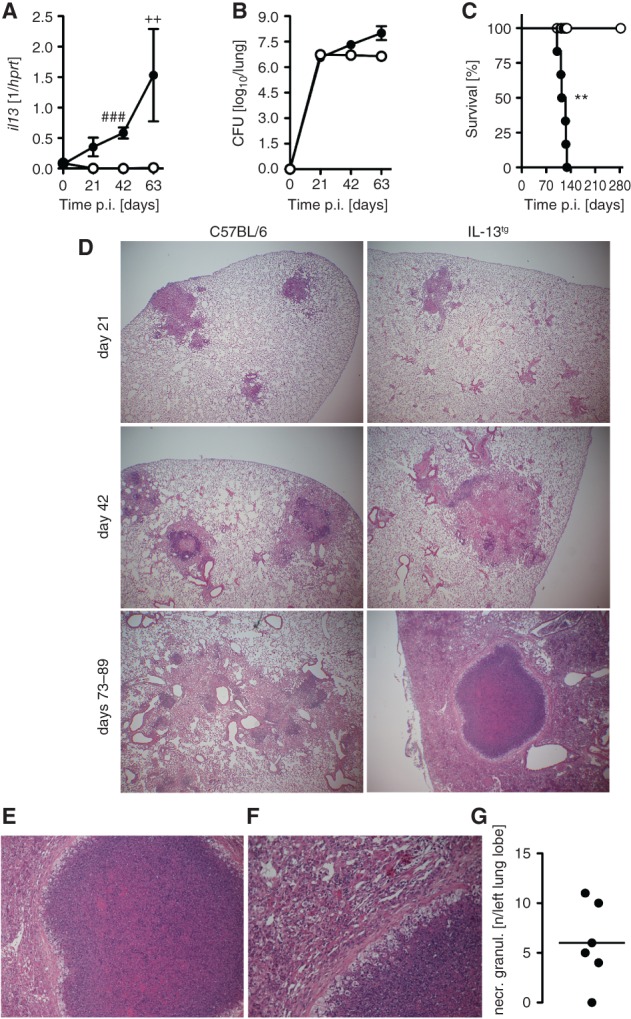
Reactivation and granuloma necrosis in *Mtb*-infected IL-13^tg^ mice. C57BL/6 (white symbols, left panel) and IL-13^tg^ (black symbols, right panel) mice were infected with 100 CFU *Mtb* H37Rv/aerosol. (A) During the course of infection, gene expression of *il13* (*n =* 5; ***p = 0.0009, ^###^*p* < 10^−3^, ^++^*p* = 0.002; unpaired *t*-test) was determined by RT–PCR. (B) At different time points, CFU were determined in lung homogenates. Data represent mean and SD (*n =* 5 mice; ^##^*p* = 0.0012, ^+++^*p* = 0.0001; unpaired *t*-test). (C) Survival of *Mtb*-infected mice (*n =* 8 mice; **p = 0.0027; log-rank test); one representative experiment of two is shown. (D–F) Granulomatous response in formalin-fixed and H&E-stained lung sections (*n =* 4–6). (D) During the course of *Mtb* infection, IL-13^tg^ mice developed extensive pulmonary inflammation and centrally necrotizing granulomas similar to the pathology observed in human TB patients; magnification = ×40. (E) In IL-13^tg^ mice, necrotic granulomas were surrounded by a fibrous layer and epithelioid macrophages; magnification = ×100. (F) Cell debris inside necrotic granuloma of an IL-13^tg^ mouse; magnification = ×200. (G) Necrotic granulomas were defined as acellular mass surrounded by a fibrous rim and were counted in serial sections of the left lung lobe (*n =* 6). In (A–F), one experiment representative of at least two performed is shown

In response to aerosol *Mtb* infection, wild-type mice did not appreciably express the IL-4R*α* ligands IL-4 or IL-13 (data not shown) and did not develop necrotizing granulomas (see supplementary material, Figure S1A). A deletion of the common IL-4R*α* subunit had no effect on bacterial loads and survival after *Mtb* infection (see supplementary material, Figure S1B, C). To analyse the influence of enhanced input via the IL-4R*α* chain after *Mtb* infection, we chose an IL-13^tg^ mouse line which expresses murine *il13* under control of the human CD2 locus control region 19. Hence, in these IL-13^tg^ mice, IL-13 is only produced by activated T cells 19. Gene expression of *il13* was comparable in lungs from uninfected wild-type and IL-13-over-expressing littermates (Figure 1A); 21 days after aerosol infection with 100 CFU *Mtb il13*, gene expression was found to be slowly increasing in lungs of IL-13^tg^ mice, whereas *il13* transcripts were hardly detectable in wild-type littermates. At 63 days of infection, pulmonary gene expression of *il13* was high in IL-13^tg^ mice (Figure 1A). Bacterial loads in the lungs of both wild-type and IL-13^tg^ mice were comparable after 21 days of infection (Figure 1B). At 42 and 63 days of infection, mycobacterial growth was more significantly increased in the lungs of IL-13^tg^ mice than in wild-type mice. As a consequence, *Mtb*-infected IL-13^tg^ mice died before day 140 (Figure 1C).

### *Mtb*-infected IL-13^tg^ mice develop centrally necrotizing granulomas strongly resembling human pathology in post-primary TB

After aerosol infection with *Mtb*, circumscript mononuclear foci developed in wild-type mice, which progressively increased in size over time but never became necrotic (Figure 1D). In IL-13^tg^ mice, early granulomas did not differ in size or cellularity from those in wild-type mice, but at day 42 of *Mtb* infection became more pronounced than in wild-type mice. At later time points, there was extensive pulmonary inflammation in which centrally necrotizing granulomas, resembling human TB lesions, were readily apparent (Figure 1E). The eosinophilic necrotic core consisted of dead and dying cells, with numerous granulocytes present, and was demarcated by a fibrous capsule-like layer surrounded by fibroblasts and epithelioid macrophages (Figure 1E, F). The diameters of the necrotic lesions measured ca. 1000 µm. A quantification of centrally necrotizing granulomas in serial sections of the left lung lobes revealed that, in five of six IL-13^tg^ mice, necrotic lesions were present between days 73 and 89 of *Mtb* infection, with an average of six necrotic granulomas/lung lobe (Figure 1G).

**Figure 2:**
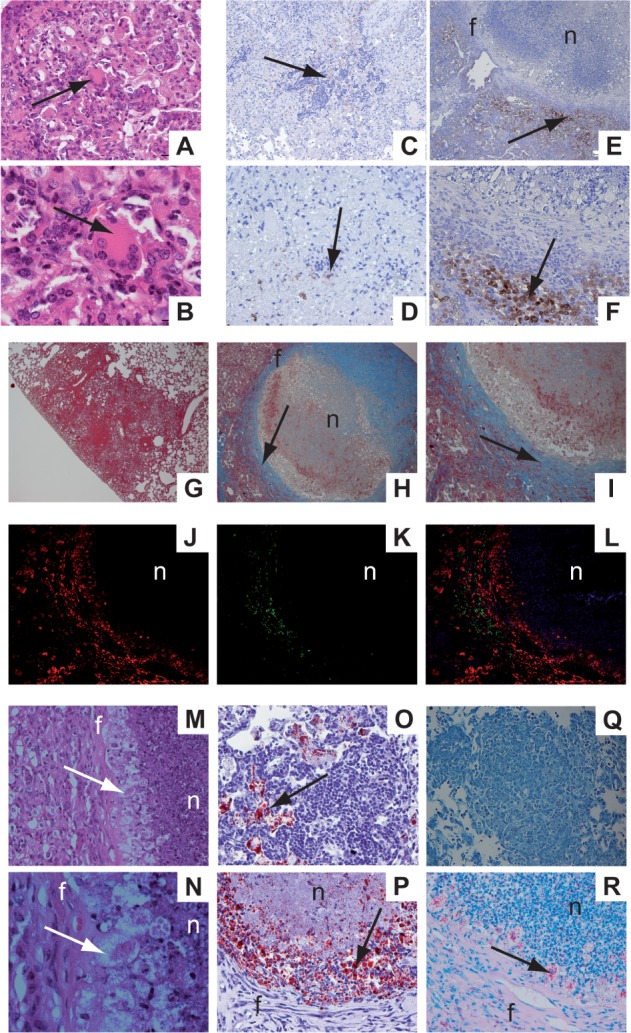
Histological characteristics of human post-primary TB in *Mtb*-infected IL-13^tg^ mice. C57BL/6 and IL-13^tg^ mice were infected with 100 CFU *Mtb* H37Rv/aerosol and lung sections were histologically evaluated between days 73 and 124 of infection. (A, B) Multinucleated giant cells (arrows) in formalin-fixed and H&E-stained lung sections of IL-13^tg^ mice (*n =* 10). (C–F) Hypoxic areas (arrows) in formalin-fixed lung sections of C57BL/6 (C, D) and IL-13^tg^ (E, F) mice after immunohistochemical detection of i.v. injected pimonidazole at a concentration of 60 mg/kg (*n =* 10). (G–I) Collagen in the fibrous rim (arrows) was stained in formalin-fixed lung section of C57BL/6 (G) and IL-13^tg^ mice (H, I) with trichrome (*n =* 10). (J–L) Immunohistochemical staining of CD68 (red staining) and CD3 (green staining) in lung cryosections of IL-13^tg^ mice (*n =* 5). (M, N) Foamy macrophages (arrow) in formalin-fixed and H&E-stained in lung sections of IL-13^tg^ mice (*n =* 7). (O, P) Lipid accumulation (arrow) in foamy macrophages after oil red staining of lung cryosections of C57BL/6 (O) and IL-13^tg^ mice (P) (*n =* 5). (Q, R) Acid-fast bacilli (arrow) in formalin-fixed lung sections of C57BL/6 (Q) and IL-13^tg^ (R) mice after ZN staining (*n =* 10). In (A–F, M, N, Q, R), representative photomicrographs of one experiment of at least two performed is shown. f, fibrous rim; n, necrosis

Characteristic features of human granulomas in TB patients are multinucleated giant cells 25, hypoxia 26, a strict stratification of a fibrous capsule that separates the necrotizing granuloma from the adjoining tissue, and foamy macrophages found adjacent to the fibrous capsule within the necrotic lesion 27. As described for human post-primary TB, the lesions of *Mtb*-infected IL-13^tg^ mice also contained multinucleated giant cells (Figure 2A, B) that were not found in infected wild-type mice (data not shown). In tumour-inoculated mice, it has been shown that after intravenous (i.v.) injection of pimonidazole and subsequent immunohistochemical analysis, tumour areas close to necrosis show marked staining, indicating reduced oxygen content 28. In the present study, lesions of wild-type mice show only a few foci positive for pimonidazole (Figure 2C, D) 28. In contrast, in lungs of infected IL-13^tg^ mice areas around necrotic granulomas were intensely hypoxic (Figure 2E, F). A histological evaluation of pulmonary lesions in *Mtb*-infected mice revealed that whereas collagen deposition in lung sections from wild-type mice was hardly detectable (Figure 2G), necrotic centres of granulomas in IL-13^tg^ mice were surrounded by collagen fibres (Figure 2H, I); this fibrous rim demarcated the necrotic area from the adjoining lung tissue. Additionally, immunohistochemical staining of CD68 and CD3 cells in these lesions showed that CD3^+^ T cells were separated from the necrotic centre by a layer of CD68^+^ macrophages (Figure 2 J–L). In *Mtb*-infected IL-13^tg^ mice, foamy macrophages were also found below the fibrous rims of necrotizing granulomas (Figure 2 M, N). In contrast to a scattered distribution of lipid-containing cells in wild-type mice (Figure 2O), oil red staining of lesions in IL-13^tg^ mice clearly showed that these macrophages between the fibrous rim and the necrotic centre were full of lipid droplets (Figure 2P) strongly resembling foamy macrophages in human granulomas 27. Moreover, detection of acid-fast bacilli revealed that this zone of foamy macrophages below the fibrous capsule of necrotizing granulomas in lungs from *Mtb*-infected IL-13^tg^ mice represented a distinct area of bacterial replication (Figure 2R). Genetic ablation of IL-4R*α* in IL-13^tg^ mice resulted in a histopathological phenotype of *Mtb*-infected wild-type mice without necrotic lesions (see supplementary material, Figure S2).

### Cell-mediated immune responses are still operative in *Mtb*-infected IL-13^tg^ mice

**Figure 3:**
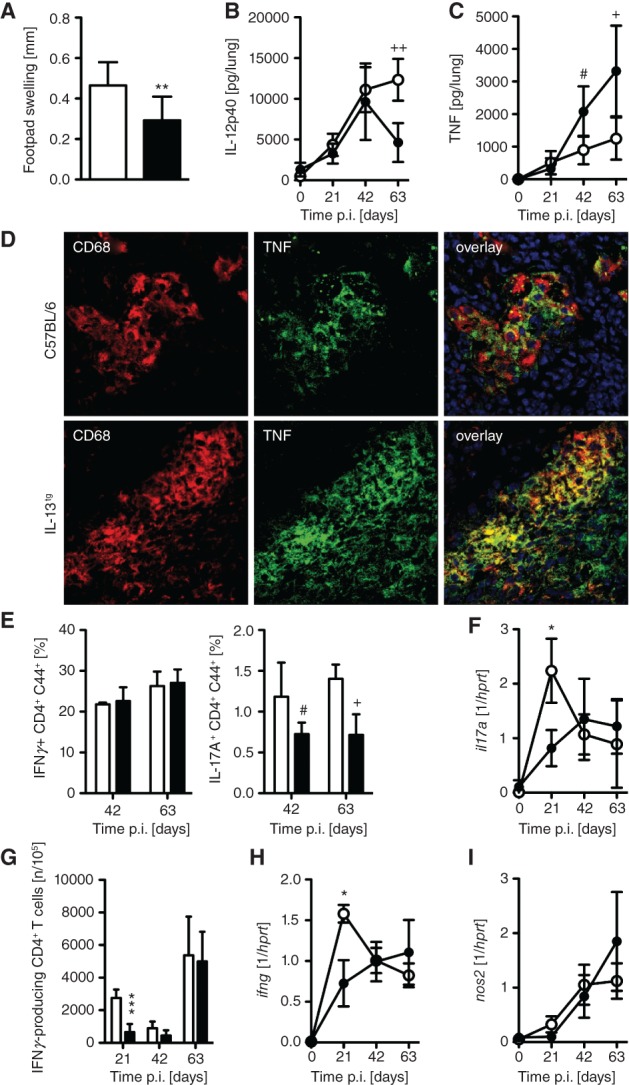
The cell-mediated immune responses in *Mtb*-infected IL-13^tg^ mice is delayed. C57BL/6 (white symbols) and IL-13^tg^ (black symbols) mice were infected with 100 CFU *Mtb* H37Rv/aerosol. (A) Antigen-specific DTH reaction after s.c. injection of PPD at 28 days after infection; results represent mean and SD (*n =* 10; **p = 0.0038; unpaired *t*-test). Before and after infection, the production of (B) IL-12p40 (*n =* 5, ^++^*p* = 0.0012; unpaired *t*-test) and (C) TNF (*n =* 5; ^#^*p* = 0.0178, ^+^*p* = 0.0164; unpaired *t*-test) in lung homogenates was determined by CBA; results represent means and SD; one experiment representative of three performed is shown. (D) Staining of CD68-positive macrophages (red) and TNF (green); *n =* 4–5; co-staining in the overlay (yellow); in IL-13^tg^ mice, TNF is expressed by CD68-positive macrophages that were found around necrotic lesions; blue, DAPI). (E) After 42 days of infection, lung cells of C57BL/6 and IL-13^tg^ mice were restimulated with anti-CD3/CD28; cells were subsequently stained extracellularly for CD90.2, CD44 and CD4, followed by intracellular staining for IFN*γ* and IL-17A, analysed by flow cytometry and the frequencies of cytokine-producing cells were compared (*n =* 4–5; ^#^*p* = 0.0317, ^+^*p* = 0.0159; Mann–Whitney U-test). (F) Gene expression of *il17a* in lung homogenates from infected mice was determined by quantitative real-time RT–PCR (*n =* 4–5; *p = 0.0159; Mann–Whitney U-test). (G) At different time points of infection, the frequency of responding lung CD4^+^ T cells was determined after restimulation with ESAT6_1–20_ in an IFN*γ* ELISPOT assay; results represent mean and SD (*n =* 4–5; ***p = 0.0002; unpaired *t*-test). (H, I) Gene expression of *ifng* (*n =* 5; *p = 0.0036; Mann–Whitney U-test) and *nos2* (*n =* 5; *p = 0.011; unpaired *t*-test) in lung homogenates from infected mice was determined by quantitative real-time RT–PCR; results represent mean and SD. In (A–C, F–I) one experiment representative of at least two performed is shown

**Figure 4:**
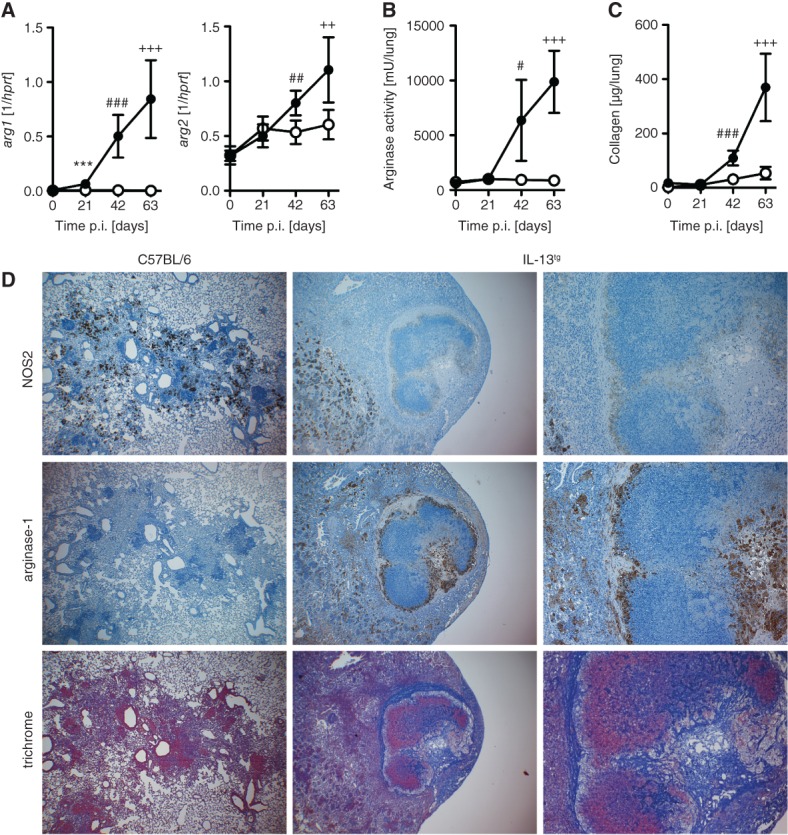
In *Mtb*-infected IL-13^tg^ mice, elevated arginase activity is accompanied by extensive collagen deposition within and around necrotic granulomas. C57BL/6 (white symbols, left panel) and IL-13^tg^ (black symbols, right panel) mice were infected with 100 CFU *Mtb* H37Rv/aerosol; At different time points: (A) gene expression of *arg1* (*n =* 5; ***p < 10^−3^, ^###^*p* = 0.0005, ^+++^*p* = 0.0008; unpaired *t*-test) and *arg2* (*n =* 5; ^##^*p* = 0.0048, ^++^*p* = 0.0090; unpaired *t*-test); (B) arginase activity (*n =* 5; ^#^*p* = 0.0112, ^+++^*p* = 0.0001; unpaired *t*-test); and (C) collagen content (*n =* 5; ^###^*p* = 0.0005, ^+++^*p* = 0.005; unpaired *t*-test) in lung homogenates from infected mice were determined; results represent mean and SD; one experiment representative of three performed is shown. (D) For immunohistological detection of NOS2 and Arg-1, lung sections were prepared from mice 73–89 days after infection (*n =* 4–6). NOS2 expression was found scattered throughout granulomas from wild-type mice; expression of the enzyme was spatially restricted in the lungs of IL-13^tg^ mice and was weak around necrotic centres. Arg-1 expression was not found in the lungs of *Mtb*-infected wild-type mice but was prominent in granulomas of IL-13^tg^ mice in a cell layer surrounding the necrotic centre. To demonstrate collagen deposition, lung sections were stained with trichrome. Collagen was moderately deposited throughout the lungs of wild-type mice, whereas it was abundant in close proximity to Arg-1-expressing cells surrounding granuloma necrotic centres in IL-13^tg^ mice. Representative results of at least two experiments are shown

Following footpad challenge with *Mtb* antigens (purified protein derivative, PPD), IL-13^tg^ mice exhibited a reduced delayed type hypersensitivity compared to wild-type mice (Figure 3A). In wild-type mice, production of IL-12/23p40 and TNF was up-regulated in lung homogenates 21, 42 and 63 days after aerosol infection with *Mtb* (Figure 3B, C). IL-13^tg^ mice expressed significantly lower levels of IL-12/23p40 than wild-type mice at day 63 (Figure 3B). In contrast, TNF production was similar in *Mtb*-infected wild-type and IL-13^tg^ mice at day 21, but was found increased in IL-13-over-expressing mice thereafter (Figure 3C). Immunofluorescence staining of CD68- and TNF-expressing cells in necrotic lesions from *Mtb*-infected IL-13^tg^ mice revealed that TNF is primarily produced by CD68-positive macrophages (Figure 3D). Additionally, these TNF-producing macrophages were located in the foamy macrophage zone below the fibrous capsule. An efficient pro-inflammatory cytokine response induces the development of protective TH1 and TH17 cells. Intracellular cytokine staining of lung CD4 T cells 42 and 63 days after *Mtb* infection revealed that the frequency of IFN*γ*-producing TH1 cells in lungs of IL-13^tg^ mice was comparable to the frequency in wild-type mice (Figure 4E). In contrast, the relative amount of IL-17A-secreting TH17 cells was reduced in lung cells of IL-13^tg^ mice (Figure 4E). However, *il-17a* gene expression was reduced in the lungs of both wild-type and IL-13^tg^ mice early after infection, but comparable thereafter (Figure 3 F). The frequency of antigen-specfic IFN*γ*-producing CD4^+^ T cells was reduced in IL-13^tg^ mice early during *Mtb* infection (Figure 3G). However, at later time points, the frequency of ELISPOT-positive cells reached identical levels to those present in infected wild-type mice. During the course of infection, *infg* and *nos2* gene expression were only reduced in lungs from IL-13^tg^ mice at 21 days of infection (Figure 3H, I).

### Enhanced alternative macrophage activation, arginase activity and collagen deposition in the lungs of *Mtb*-infected IL-13^tg^ mice

IL-4R*α*-mediated signals have been shown to induce alternatively activated macrophages (aaM*ϕ*), which develop impaired effector mechanisms against intracellular pathogens 29,30. Gene expression of *fizz1* and *ym1*, two prototype markers of alternative macrophage activation, was induced in IL-13^tg^ mice to very high levels during the whole course of infection with *Mtb* (see supplementary material, Figure S3). In contrast, *fizz1* was hardly detectable and gene expression of *ym1* was only moderately induced in lung homogenates of wild-type mice 21, 42 and 63 days after *Mtb* infection. Thus, infection of IL-13^tg^ mice with *Mtb* was associated with the presence of aaM*ϕ*.

In addition to *fizz1* and *ym1*, IL-4R*α*-mediated signals have been shown to induce Arg-1-expressing aaM*ϕ*, which develop impaired effector mechanisms against intracellular pathogens such as *Mtb* 30–32. Moreover, Arg-1 expression has been recently described in lesions from TB patients 33. After aerosol infection with *Mtb, arg1* mRNA was hardly detectable in wild-type mice (Figure 4A). Compared with this, *arg1* expression was significantly elevated in lungs from infected IL-13^tg^ mice 21, 42 and 63 days after infection. In addition to *arg1*, gene expression of *arg2* were also found to be increased on days 42 and 63 of *Mtb* infection in lung homogenates from IL-13^tg^ mice (Figure 4A). In lungs of uninfected mice, arginase enzyme activity was not detectable (Figure 4B). After infection with *Mtb*, only low activity was detectable in lung homogenates from wild-type mice. In IL-13^tg^ mice, arginase activity in lung homogenates increased steadily and maintained significantly elevated levels throughout the infection. At days 42 and 63 of infection, collagen deposition was significantly increased in lung homogenates from IL-13^tg^ mice when compared to wild-type mice (Figure 4C).

Differential expression of NOS2 and Arg-1 is important for regulating macrophage effector functions and tissue remodelling 29,30. Between 73 and 89 days after infection with *Mtb*, NOS2 was expressed throughout the granulomas in both wild-type and IL-13^tg^ mice, with the exception of necrotic granuloma centres (Figure 4D). In contrast, Arg-1 expression was undetectable in the lungs of *Mtb*-infected wild-type mice but was very prominent in lung granulomas of IL-13^tg^ mice (Figure 4D). Here, necrotic centres of the granulomas were typically surrounded by Arg-1-expressing cells.

Because l-ornithine, a product of arginase activity, is a necessary metabolite for the production of proline, which in turn controls collagen production, arginase activity has also been linked to tissue remodelling and fibrosis 29. In human TB, peripheral blood mononuclear cells from patients with pulmonary disease display higher arginase activity 34 and progressive necrotic lesions are walled off by fibrotic tissue 2, suggesting that arginase activity may contribute to pathology. We therefore analysed collagen deposition in lungs from *Mtb*-infected mice. Whereas collagen was only weakly produced and diffusely deposited in the lung interstitium of wild-type mice 63 days after infection with *Mtb*, collagen was deposited as a dense, capsule-like zone, mainly around pulmonary granulomas, in IL-13^tg^ mice (Figure 4D). Collagen was generally found in close proximity to the zone of Arg-1-expressing cells.

**Figure 5:**
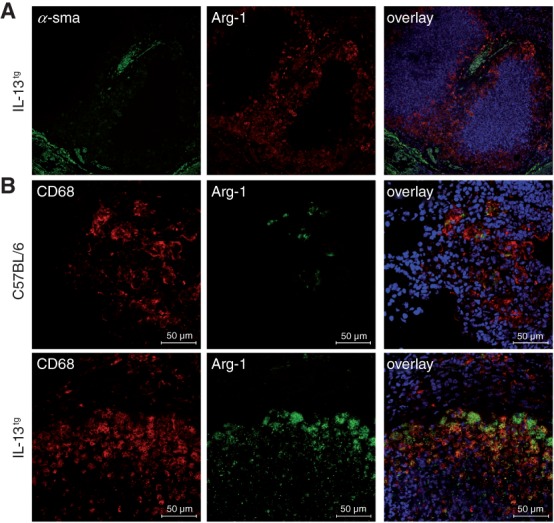
Macrophages around necrotizing granulomatous lesions produce Arg-1; IL-13^tg^ mice were infected with 100 CFU *Mtb* H37Rv/aerosol. For immunofluorescent staining, lung sections were prepared from mice 81–124 days after infection: (A) staining of sma-positive (green) myofibroblasts and Arg-1 (red) (*n =* 10; no co-staining in the overlay: sma-positive cells did not produce Arg-1; blue, DAPI); (B) staining of CD68-positive macrophages (red) and Arg-1 (green); *n =* 10; co-staining in the overlay (yellow): in IL-13^tg^ mice, Arg-1 is expressed by CD68-positive macrophages that were found around necrotic lesions; blue, DAPI

It is well documented that IL-13 preferentially activates Arg-1 and production of l-ornithine in macrophages, but it may also act on fibroblasts and myofibroblasts 35. However, co-staining of Arg-1 and *α*-sma in lungs from *Mtb*-infected IL-13^tg^ mice showed no Arg-1 expression in the latter (Figure 5A). In contrast, immunofluorescence staining of Arg-1- and CD68-expressing cells in necrotic lesions from *Mtb*-infected IL-13^tg^ mice revealed that Arg-1 is primarily produced by CD68-positive macrophages (Figure 5B). Additionally, these arginase-producing macrophages were located in the foamy macrophage zone below the fibrous capsule.

### In *Mtb*-infected wild-type mice, increased arginase activity after NOS2 inhibition was associated with GN

**Figure 6:**
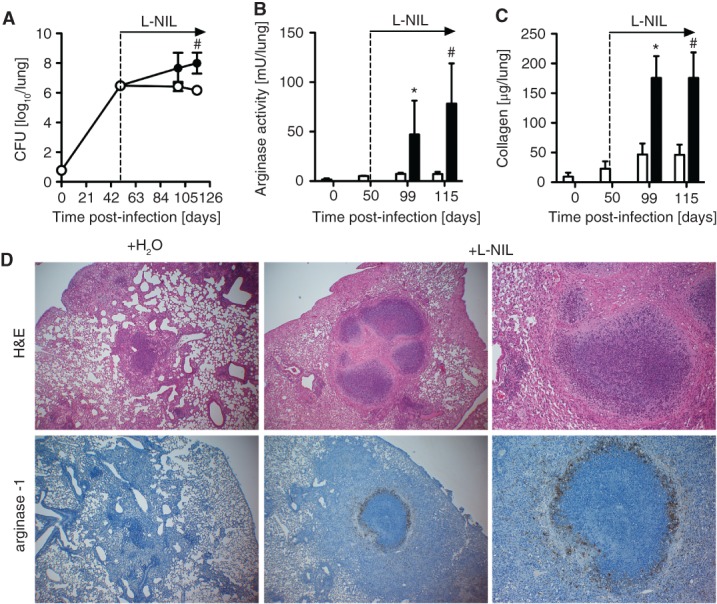
Induction of arginase activity in *Mtb*-infected wild-type mice leads to recrudescent *Mtb* growth and granuloma necrosis. (A–D) NOS2 activity was blocked by treating C57BL/6 49 days after aerosol infection with 100 CFU *Mtb*, with L-NIL administered at 10 mm in drinking water (black symbols); infected control mice received untreated water (white symbols). (A) At different time points, CFU in lungs were determined in lung homogenates; data represent mean and SD (*n =* 3–4 mice; ^#^*p* = 0.0286; Mann–Whitney test). (B) Arginase activity (*n =* 4; *p = 0.0286, ^#^*p* = 0.0286; Mann–Whitney test) and (C) collagen content (*n =* 4; *p = 0.0286; Mann–Whitney test) in lung homogenates from infected untreated and L-NIL treated mice were determined; data represent mean and SD. (D) Granulomatous response and Arg-1 expression in formalin-fixed lung sections from untreated (left panel) and L-NIL treated mice (right panel) 100 days after *Mtb* infection (*n =* 4). NOS inhibition resulted in increased pulmonary inflammation and the appearance of centrally necrotizing granulomas; magnification = ×40. Necrotic granuloma centres contained cellular debris and were surrounded by a fibrous capsule; magnification = ×100). Arg-1 expression was induced in L-NIL-treated mice (magnification = ×40) and necrotizing granulomas were surrounded by a zone of Arg-1-expressing cells (magnification = ×200). In (A–D) one experiment representative of three performed is shown

To provide further support for our hypothesis that Arg-1 induction may be involved in recrudescent *Mtb* growth and GN, we sought to increase Arg-1 expression in wild-type mice independently of the IL-13/IL-4R*α* pathway. Because Arg-1 expression is endogenously controlled by the NOS2-dependent production of l-hydroxyarginine 29, we examined the effect of inhibiting NOS2 on Arg-1 expression, mycobacterial growth and *Mtb*-associated tissue pathology in wild-type mice as an alternative strategy. As previously shown 36, NOS2 inhibition by the treatment with L-NIL significantly increased bacterial loads in the lungs of infected animals (Figure 6A). NOS2 inhibition resulted in significantly increased arginase activity and extensive collagen deposition in the lungs (Figure 6B, C). This was accompanied by the development of centrally necrotizing granulomas in the lungs of L-NIL-treated mice, surrounded by Arg-1-expressing cells (Figure 6D), very similar to the pathology observed in *Mtb*-infected IL-13^tg^ mice.

## Discussion

A role for IL-4R*α*-driven TH2 immunity in TB disease exacerbation has been postulated, primarily based on cytokine measurements in TB patients 14. Particularly, *il4* gene expression in peripheral blood mononuclear or bronchoalveolar lavage cells was often shown to be increased in TB patients 15,16,37–46 and was associated with GN and cavity formation 47. The experimental model of high-dose *Mtb* infection in BALB/c mice has been extensively used to analyse the contribution of a TH2 immune response on disease progression and has clearly identified IL-4 to be involved in focal necrosis and fibrosis 8,9,11. However, a direct influence of TH2 cytokines on the development of centrally necrotizing granulomas, the typical pathology of post-primary TB, has never been shown. Genetically resistant mice do not represent a suitable model to analyse potential IL-4R*α*-dependent mechanisms because, in response to low-dose aerosol *Mtb* infection, wild-type and IL-4R*α* mice did not develop centrally necrotizing granulomas. In the present study, however, over-expression of IL-13 in fact led to recrudescent *Mtb* growth accompanied by centrally necrotizing granulomas. Hence, IL-13/IL-4R*α*-driven mechanisms are directly linked to the development of central GN.

In addition to GN, *Mtb*-infected IL-13^tg^ mice displayed many more features of the pathology in human post-primary TB that are apparently not detectable in wild-type mice. A key attribute of lesions in post-primary TB that we have also found in *Mtb*-infected IL-13^tg^ mice is hypoxia 48, which has a great impact on gene expression and the metabolic activity of *Mtb* within lesions of infected individuals 26. Another characteristic of human post-primary TB that was also present in *Mtb*-infected IL-13^tg^ mice was a stringent stratification of necrotizing granulomas, in which the necrotic centre is surrounded by a macrophage layer and a collagen capsule that separates the granuloma from the adjoining tissue. Within the lesion, macrophages accumulate lipids secreted by replicating mycobacteria 27,49. These foamy macrophages have been shown to produce anti-inflammatory cytokines 9 and to not express NOS2 under TH2 conditions after high-dose *Mtb* infection 50. In granulomas from TB patients and *Mtb*-infected IL-13^tg^ mice, a zone of lipid-rich, acid-fast bacilli-containing foamy macrophages are found adjacent to the fibrous capsule within the necrotic lesion 27. Together, the pathology observed in *Mtb*-infected IL-13^tg^ mice, which was entirely dependent on signals transduced via IL-4R*α*, displayed many features of human post-primary TB. However, in contrast to the pathogenesis in humans, post-primary TB *Mtb* infection in IL-13^tg^ mice: (a) apparently does not reactivate from a latent, dormant phase of infection; and (b) does not result in cavity formation.

In our animal study, IL-13 only moderately influenced the induction of protective immune functions mediated by CD4^+^ T cells. However, over-expression of IL-13 led to a pronounced development of aaM*ϕ*. Importantly, *Mtb*-infected IL-13^tg^ mice had strikingly elevated expression and activity of Arg-1. Moreover, increasing enzyme activity by indirectly suppressing the production of the endogenous arginase inhibitor l-hydroxyarginine in wild-type mice was associated with the development of GN independently of the IL-13/IL-4R*α* axis. Therefore, our study suggests that increased arginase activity is involved in mediating GN in mice.

Specific elimination of Arg-1 in macrophages was recently found to enhance anti-mycobacterial effector mechanisms in macrophages and to decreased lung bacterial loads during *Mtb* infection 24,32. This fits our interpretation that enhanced enzyme activity can subvert the host immune response during TB. Because increased resistance of *Mtb*-infected, macrophage-specific Arg-1^–/–^ mice is associated with elevated amounts of NOS2-depedent production of antimycobacterial reactive nitrogen intermediates 32, depletion of the common substrate l-arginine may be a major mechanism responsible for unrestrained bacterial replication in IL-13^tg^ mice. Additionally, as has been shown for other intracellular pathogens, the Arg-1-dependent production of polyamines may have directly promoted bacterial growth in *Mtb*-infected IL-13^tg^ mice 51. Arginase activity has also been linked to tissue remodelling and fibrosis 29. In human TB, patients with pulmonary disease display higher arginase activity 34, and progressive necrotic lesions are walled off by fibrotic tissue 2, suggesting that arginase activity not only promotes bacterial replication but may also contribute to pathology via collagen production.

Other mechanisms may also mediate GN. In a TH2 environment, TNF has exacerbated cytotoxic effects 52. Therefore, the increased expression of TNF by macrophages below the fibrous rims of granulomas in *Mtb*-infected IL-13^tg^ mice might also contribute to central GN. It is additionally possible that enhanced *Mtb* replication is, *per se*, a determinant of cell death; alternative macrophage activation via the IL-4/IL-13 receptor would thus cause central GN, mainly because the resumption of *Mtb* replication at the centres of granulomas would destroy *Mtb*-harbouring lipid-rich foamy macrophages. In this regard, trehalose 6,6′-dimycolate (TDM), a toxic lipid extractable from the surface of virulent *Mtb*, has been shown to contribute to the pathogenesis of caseating granulomas in a lipid-rich milieu 53. Moreover, in the experimental model of high-dose *Mtb* infection of BALB/c mice, the accumulation of foamy macrophages has been associated with increased mycobacterial replication and tissue damage after infection with a highly virulent Beijing strain 54. Together, alternative macrophage activation and Arg-1 activity may promote *Mtb* growth in the protected environment of a collagen capsule that may accumulate lipids and TDM in foamy macrophages, thereby initiating GN in post-primary TB.

Based on comparative histopathology in IL-13^tg^ and other immunodeficient mice infected with *Mtb* (data not shown), we favour the interpretation that additional, *Mtb* replication-independent, effects of IL-4R*α*-mediated macrophage activation are critical determinants of the observed characteristic form of central granuloma caseation, that differs from widespread, malorganized tissue necrosis occurring during primary TB in immunodeficient hosts. However, in a model of *Mtb*-induced tissue pathology in mice lacking the genetic locus *sst1*, it was demonstrated that GN may occur in the absence of dramatic changes in *Mtb* replication 55. B6.C3H-sst1 mice are deficient in *ipr1*, which appears to be an adaptor protein that may be involved in macrophage responsiveness to type I and II interferons 55. We did not find differential gene expression of *ipr1* in wild-type and IL-13^tg^ mice (data not shown). In another model, in which IFN*γ*-depleted NOS2^–/–^ mice were intradermally infected with *Mtb*, enhanced expression of cathepsin G also led to GN 56. In IL-13^tg^ mice, cathepsin G gene expression was not altered after infection with *Mtb* (data not shown). Arginase activity has been shown to be elevated in NOS2^–/–^ mice 29, but Arg-1 expression was apparently not analysed after dermal *Mtb* infection 56. An unrestricted arginase activity may, however, represent a common mechanism to be involved in driving GN in *Mtb*-infected NOS2^–/–^- 56 and L-NIL-treated or IL-13^tg^ (this study) mice.

Together, our data reinforce previous studies 8–11,13,50,54,57 that revealed an impact of TH2 immune responses on disease progression in *Mtb*-infected mice. Most importantly, the present study showed IL-13 over-expression to cause recrudescent *Mtb* replication and centrally necrotizing granulomas in experimental TB, strongly resembling the pathology of human TB. Together, it is worth considering that *Mtb* may have developed a strategy to hijack the IL-13/IL-4R*α* axis in order to: (a) promote its own survival within the host; and (b) facilitate an exit route by driving GN necessary for endobronchial dissemination and spread into the environment. Targeting IL-4R*α* downstream mechanisms may, therefore, represent an adjunctive approach to treat or prevent post-primary TB. The mouse model described here may be a useful tool in validating such strategies.

## SUPPLEMENTARY MATERIAL ON THE INTERNET

The following supplementary material may be found in the online version of this article:

**Figure S1.** No role of IL-4 and IL-13 in *Mtb*-infected wild-type mice for protective immune responses and pathology

**Figure S2.** No granuloma necrosis in IL-4R*α*-deficient *Mtb*-infected IL-13^tg^ mice

**Figure S3.** Over-expression of IL-13 results in alternative macrophage activation after infection with *Mtb*

## Supporting Information

No role of IL-4 and IL-13 in *Mtb*-infected wild-type mice for protective immune responses and pathology. C57BL/6 (white symbols) or IL-4R*α*^–/–^ (grey symbols) mice were infected with 100 CFU *Mtb* H37Rv/aerosol. (A) Moribund *MTb*-infected mice developed advanced inflammatory cell infiltration, interstitial fibrosis and deposition of cholesterol crystals without developing centrally necrotizing granulomas (magnification = ×40); representative photomicrographs of four mice are shown. (B) At different time points, CFU were determined in lung homogenates; data represent mean and standard deviation (*n =* 4 mice); one experiment representative of three performed is shown. (C) Survival of *Mtb*-infected mice (*n =* 14 mice). In (A, B) one experiment representative of two is shown

No granuloma necrosis in IL-4R*α*-deficient *Mtb*-infected IL-13^tg^ mice. BALB/c (left panel), IL-13^tg^ (middle panel) and IL-13^tg^ × IL-4R*α*^–/–^ (right panel) mice were infected with 100 CFU *Mtb* H37Rv/aerosol. After 113 days the granulomatous response was evaluated in formalin-fixed and haematoxylin and eosin-stained lung sections (*n* = 4–5). Granuloma necrosis was found in infected IL-13^tg^ mice but was absent in BALB/c and IL-13^tg^ × IL-4R*α*^–/–^ mice. Representative photomicrographs of one experiment of two performed are shown

Over-expression of IL-13 results in alternative macrophage activation after infection with *Mtb*. C57BL/6 (white symbols, solid line) and IL-13^tg^ (black symbols, dotted line) mice were infected with 100 CFU *Mtb* H37Rv/aerosol. At different time points, gene expressions of (A) *fizz1* (*n* = 5; ****p* < 10^−3^, ^###^*p* < 10^−3^, ^+++^*p* < 10^−3^; unpaired *t*-test) and (B) *ym1* (*n* = 5; ****p* = 0.0062, ^###^*p* = 0033, ^+++^*p* < 10^−3^; unpaired *t*-test) in lung homogenates of infected mice were determined by quantitative real-time RT–PCR, based on gene expression of *hprt*. Results represent mean and SD. One experiment representative of two performed is shown
