# Supplementary figures and images for: The IL-13/IL-4Rα axis is involved in tuberculosis-associated pathology
Source: J Pathol. 2014 Aug 6;234(3):338–50. doi: 10.1002/path.4399 (PMC4277691; doi:10.1002/path.4399)

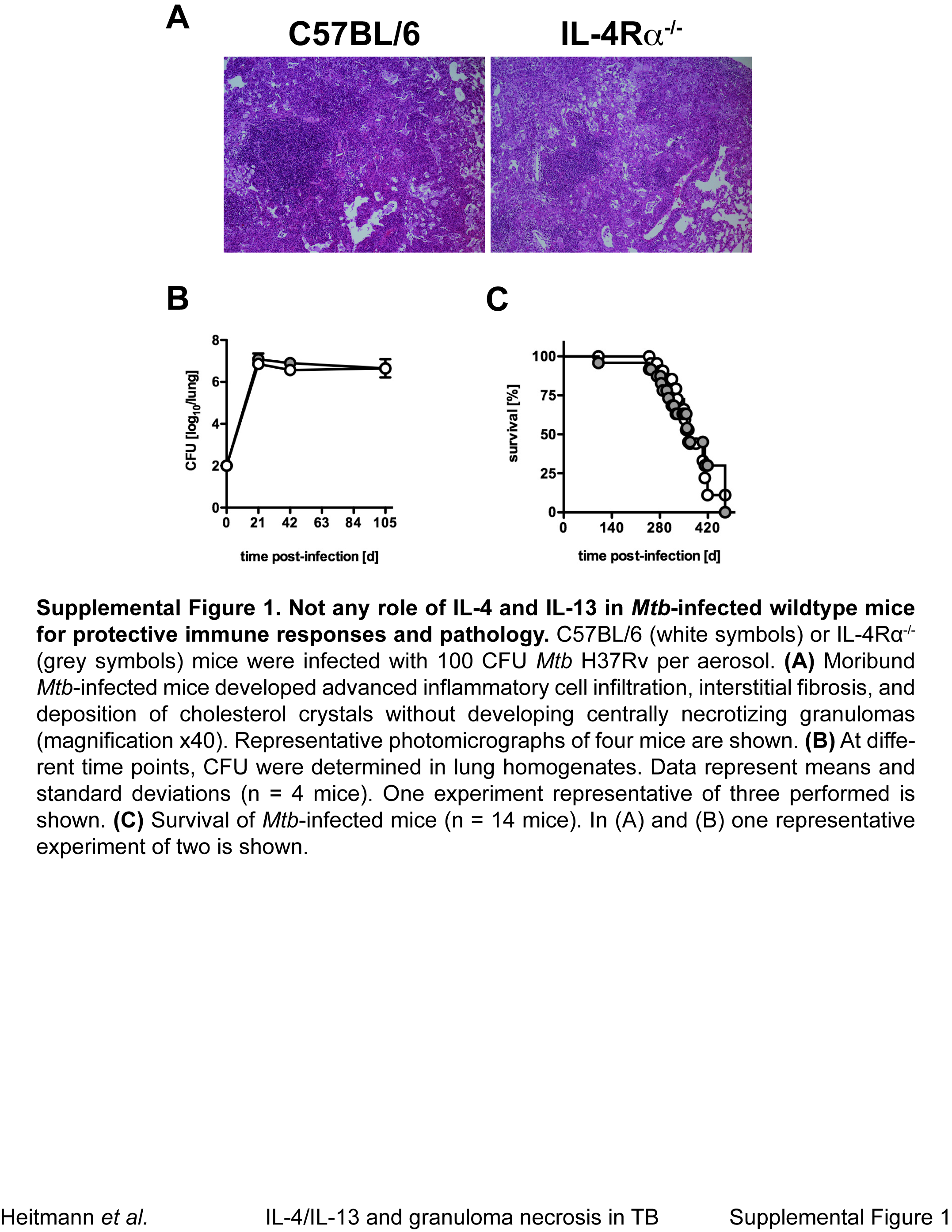

Supplement: Supplementary file 1 — No role of IL-4 and IL-13 in Mtb-infected wild-type mice for protective immune responses and pathology. C57BL/6 (white symbols) or IL-4Rα–/– (grey symbols) mice were infected with 100 CFU Mtb H37Rv/aerosol. (A) Moribund MTb-infected mice developed advanced inflammatory cell infiltration, interstitial fibrosis and deposition of cholesterol crystals without developing centrally necrotizing granulomas (magnification = ×40); representative photomicrographs of four mice are shown. (B) At different time points, CFU were determined in lung homogenates; data represent mean and standard deviation (n = 4 mice); one experiment representative of three performed is shown. (C) Survival of Mtb-infected mice (n = 14 mice). In (A, B) one experiment representative of two is shown [file path0234-0338-sd1.tif]

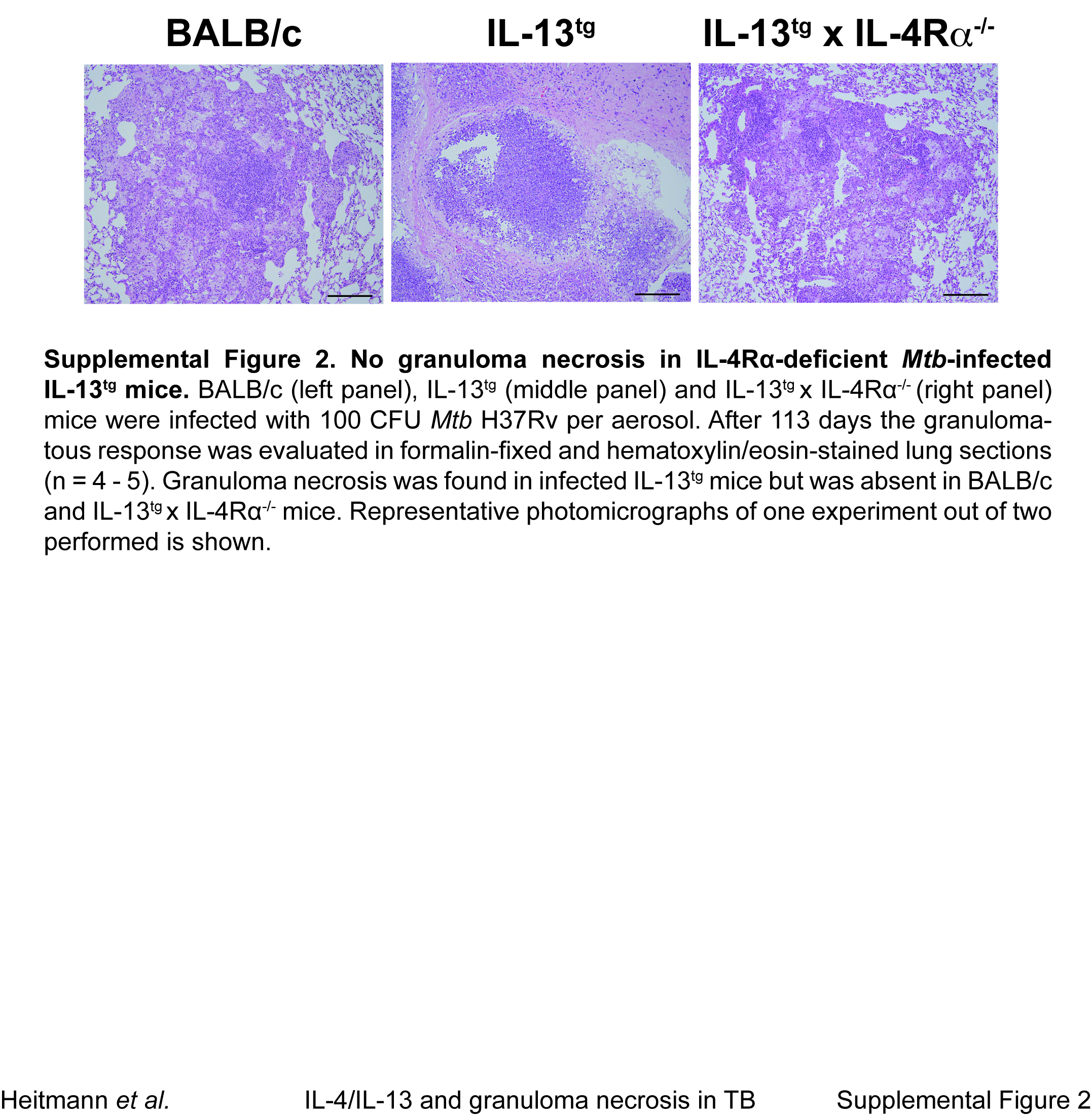

Supplement: Supplementary file 2 — No granuloma necrosis in IL-4Rα-deficient Mtb-infected IL-13tg mice. BALB/c (left panel), IL-13tg (middle panel) and IL-13tg × IL-4Rα–/– (right panel) mice were infected with 100 CFU Mtb H37Rv/aerosol. After 113 days the granulomatous response was evaluated in formalin-fixed and haematoxylin and eosin-stained lung sections (n = 4–5). Granuloma necrosis was found in infected IL-13tg mice but was absent in BALB/c and IL-13tg × IL-4Rα–/– mice. Representative photomicrographs of one experiment of two performed are shown [file path0234-0338-sd2.tif]

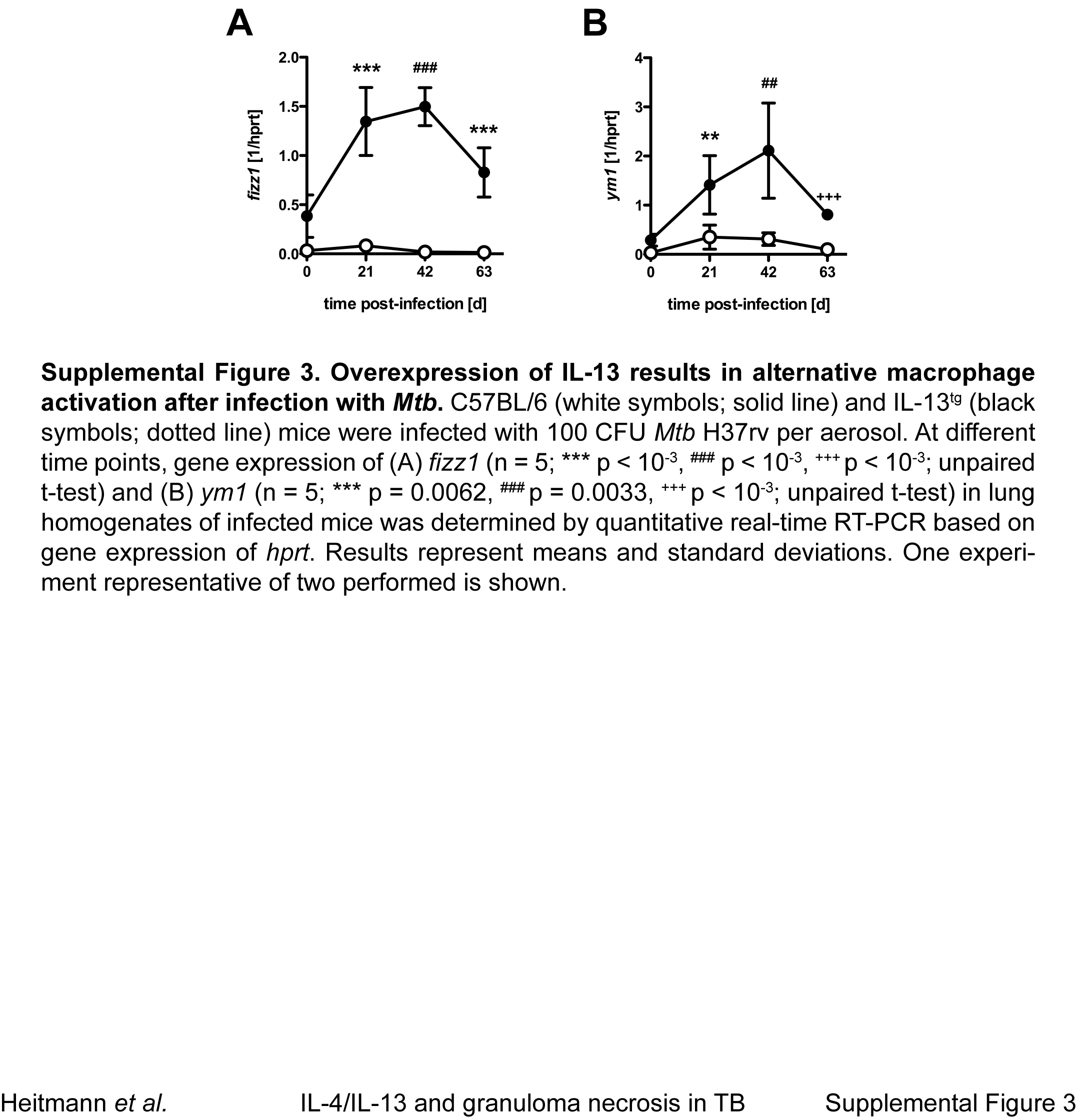

Supplement: Supplementary file 3 — Over-expression of IL-13 results in alternative macrophage activation after infection with Mtb. C57BL/6 (white symbols, solid line) and IL-13tg (black symbols, dotted line) mice were infected with 100 CFU Mtb H37Rv/aerosol. At different time points, gene expressions of (A) fizz1 (n = 5; ***p < 10−3, ###p < 10−3, +++p < 10−3; unpaired t-test) and (B) ym1 (n = 5; ***p = 0.0062, ###p = 0033, +++p < 10−3; unpaired t-test) in lung homogenates of infected mice were determined by quantitative real-time RT–PCR, based on gene expression of hprt. Results represent mean and SD. One experiment representative of two performed is shown [file path0234-0338-sd3.tif]
